# Supplementary material for: Dopaminergic processes predict temporal distortions in event memory
Source: Nat Commun. 2026 Mar 14;17:3971. doi: 10.1038/s41467-026-69950-8 (PMC13133266; doi:10.1038/s41467-026-69950-8)
Supplement: Supplementary file 1 — Supplementary Information [file 41467_2026_69950_MOESM1_ESM.pdf]

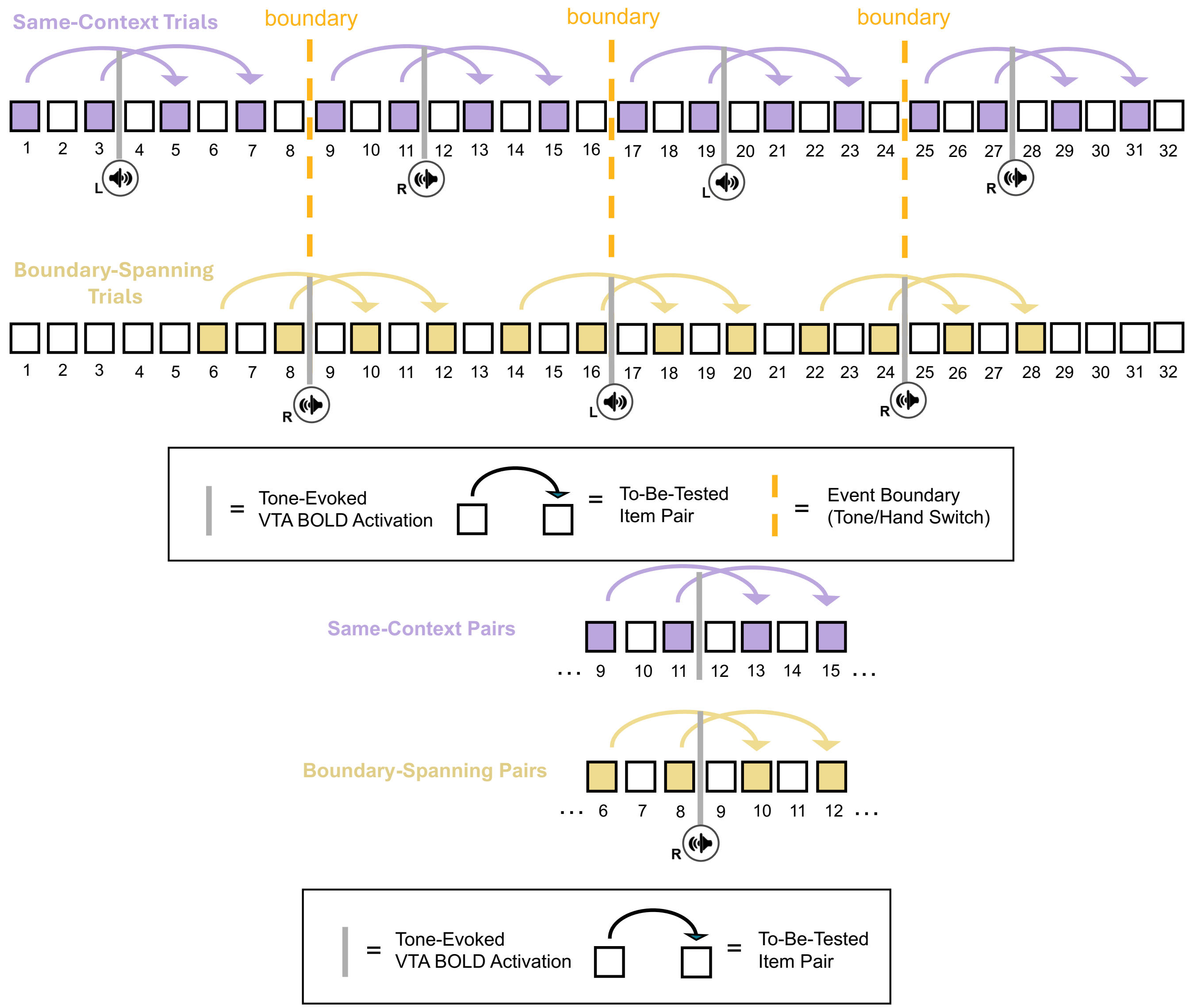

Supplementary Figure 1. Item pair structure across the event sequence task. (Top panel) Each sequence contained 32 images of everyday objects. A pure tone was played either in participants' left or right ear for 8 successive items. It then switched to the other ear and changed in pitch. This pattern continued until the end of the sequence. Colored squares denote the item pair positions that were subsequently queried during the temporal memory tests after each list. The arrow connections indicate which item pairs were tested together. These pairs were always three intervening items apart during encoding. Purple squares show the pair positions for same-context trials, or to-be-tested item pairs that were presented with the same tone. Yellow squares show the pair positions for boundary trials, or to-be-tested item pairs that spanned an intervening tone switch. Vertical dashed yellow lines indicate the positions of event boundaries, or the three tone switches in each list. For the modeling analyses, we aimed to relate trial-level estimates of tone-evoked VTA activation to both temporal distance memory ratings and blinking associated with their corresponding item pairs. To align these two measures, we specifically focused on VTA parameter estimates evoked by event boundaries and same-context tones position-matched to those locations (all vertical gray lines). (Bottom panel) Example of position-matching between tone-evoked VTA activation between conditions. This matching provided a critical reference point for controlling timing-related effects of VTA activation relative to the positioning of the to-be-tested item pairs spanning event boundaries. In this example section, the first boundary tone occurred after the 8th item in the list. This means that the boundary occurred two items after the first

item in the first boundary-spanning pair (i.e., item in position 6), and immediately after the first item in the second boundary-spanning item pair (i.e., item in position 8). To position-match these timing effects in the same-context condition, the two same-context pairs were also temporally aligned with the same sampling points. That is, the relative positioning of the selected tones in the analysis was the same as the two boundary-spanning pairs for each event boundary in the list.

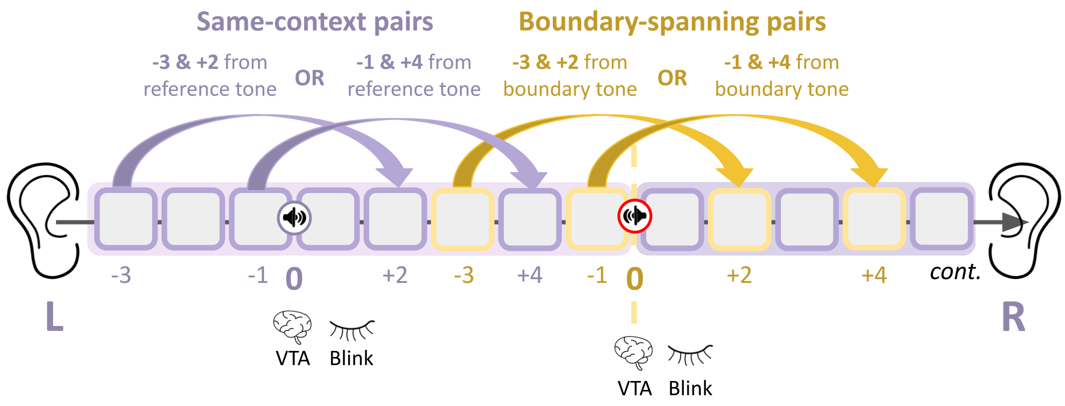

Supplementary Figure 2. Detailed schematic of position labeling and matching between conditions. Several analyses examined how indirect measures of brief dopaminergic processes during encoding (i.e., tone-related VTA activation or post-tone blink count) predicted larger-scale outcomes (i.e., memory for temporal distance or blink rate between to-be-tested pairs). For these analyses, one intervening tone was selected for each to-be-tested pair in the event encoding sequence. Arrow connections represent to-be-tested pairs, including same-context pairs (purple) and boundary-spanning pairs (yellow). For boundary-spanning pairs, the selected tone was the tone switch that denoted an event boundary (red circle). Depending on the particular boundary-spanning pair, the to-be-tested items fell either 3 positions before (-3) and 2 positions after (+2) the tone switch or 1 position before (-1) and 4 positions after (+4) the tone switch. For same-context pairs, the selected tone (purple circle) matched these positions (i.e., -3 and +2 or -1 and +4).

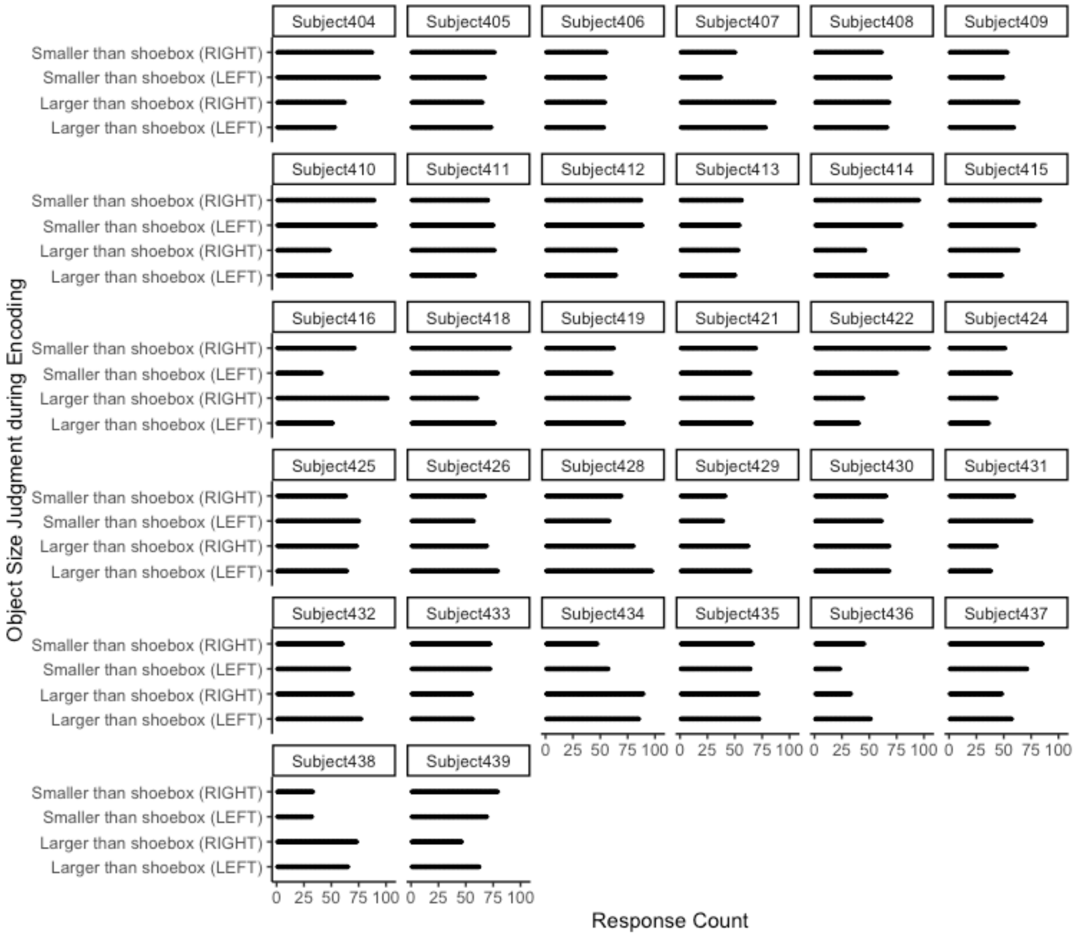

Supplementary Figure 3. All participants used all four response options during the encoding orienting question. Response distributions for each participant to the simple orienting question: Is this object larger or smaller than a standard shoebox?. For each object image, participants were instructed to respond with a button box in their left or right hand, depending on the side of the preceding auditory tone (e.g., right ear = right hand). Source data are provided as a Source Data file.

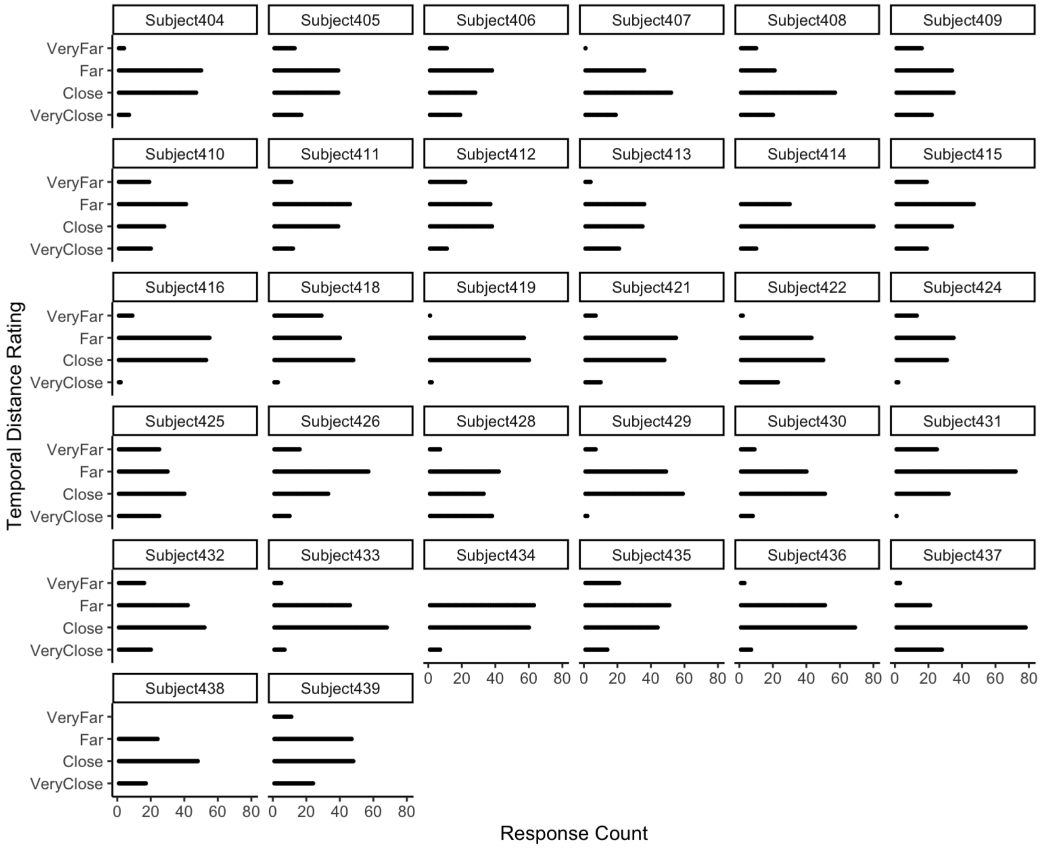

Supplementary Figure 4. Participants generally used all four rating options on the temporal distance memory test. Response distributions for each participant on the temporal distance memory test. On each test trial, participants were presented with different pairs of objects from the prior sequence and asked to rate how far apart they thought each pair appeared in time (very close, close, far, or very far), despite their equivalent objective distance during encoding. Source data are provided as a Source Data file.

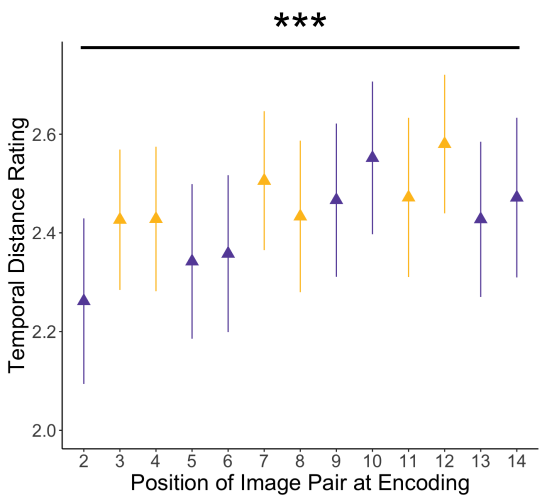

Supplementary Figure 5. Later temporal distance memory expands over an entire encoding block. Mean ratings on the temporal distance memory test by position of the to-be-tested image pairs across an entire encoding block. Ratings (1-4) are displayed as a continuous variable. Yellow triangles represent boundary-spanning item pairs, while purple triangles represent same-context item pairs. In order of pair presentation at encoding, the boundary-spanning pairs were the 3rd, 4th, 7th, 8th, 11th, and 12th item pairs. Error bars represent SEM. $n = 32$ participants. Statistical significance refers to results from trial-level cumulative link model in which pair position was modeled as an integer fixed-effect predictor of temporal distance ratings ($z = 4.31$, $p = .000016$; odds ratio = 1.04, 95% CI of odds ratio = [1.02, 1.05]). ***$p < .001$. Source data are provided as a Source Data file.

Relationships between noradrenergic and dopaminergic system activation measures and other critical temporal distance and eye-tracking results. According to theoretical frameworks of event segmentation (Zacks & Sargent, 2010), both the dopaminergic and noradrenergic systems are well positioned to elicit a global updating signal when event transitions occur. However, it is possible that they contribute to memory separation and blink behavior in different ways (Rouhani et al., 2024). We have also previously shown that LC activation at boundaries relates to impairments in temporal order memory, another index of memory separation, and more differentiated multivoxel activation patterns in left dentate gyrus (DG), a hippocampal subregion that is important for memory encoding and in disambiguating overlapping representations (Clewett et al., 2025). Much work shows that dopaminergic modulation of hippocampal processing also plays an essential role in structuring and encoding episodic memories, making it another candidate input signal for memory separation at its target regions (Shohamy & Adcock, 2010). In the next set of exploratory analyses, we sought to determine potential differences and similarities between these systems' contributions to temporal memory and links to different eye-tracking measures.

Locus coeruleus (LC) region-of-interest (ROI) definition. The anatomy of the LC was visualized in each participant using a fast spin echo (FSE) T1 MRI sequence, which is sensitive to neuromelanin and water content within LC neurons. Each participant's left and right LC were hand-drawn separately by trained raters and then transformed into each participant's run-level functional space to extract parameter estimates of trial-level LC activation. For more details about this MRI sequence, drawing procedures, and analysis methods, see Clewett et al. (2025).

Testing the relationship between tone-related LC activation and temporal distance ratings. To assess whether temporal distance memory could also be predicted by LC activation, we fit a cumulative link model where tone-related LC activation was mean-centered by participant and modeled as a fixed-effect predictor. Condition was modeled as a categorical variable, and we also included an interaction term between the two. Pair position at encoding was modeled as an integer fixed-effect predictor. Participant ID was modeled as a random effect. The results revealed no significant main effect of LC activation on temporal distance ratings (z = .59, p = .56; odds ratio = 1.02, 95% CI of odds ratio = [.96, 1.08]) nor significant LC-by-pair type interaction effect (z = .58, p = .56; odds ratio = 1.02, 95% CI of odds ratio = [.96, 1.08]) (see **Supplementary Figure 6** below for results for each pair type separately).

Comparing relationships between tone-related LC vs. VTA activation and distance memory. Next, we clarified whether the coupling between brainstem activation and temporal distance memory for boundary pairs was specific to the VTA. We performed a likelihood-ratio test that compared a full model with both VTA and LC activation as predictors with a restricted model that averaged VTA and LC activation as one predictor. The test revealed that the full model did not significantly improve fit over the restricted model ($\chi^2(1)$ = .83, p = .36). Therefore, we cannot conclude that the relationship with temporal distance for boundary pairs is unique to VTA activation.

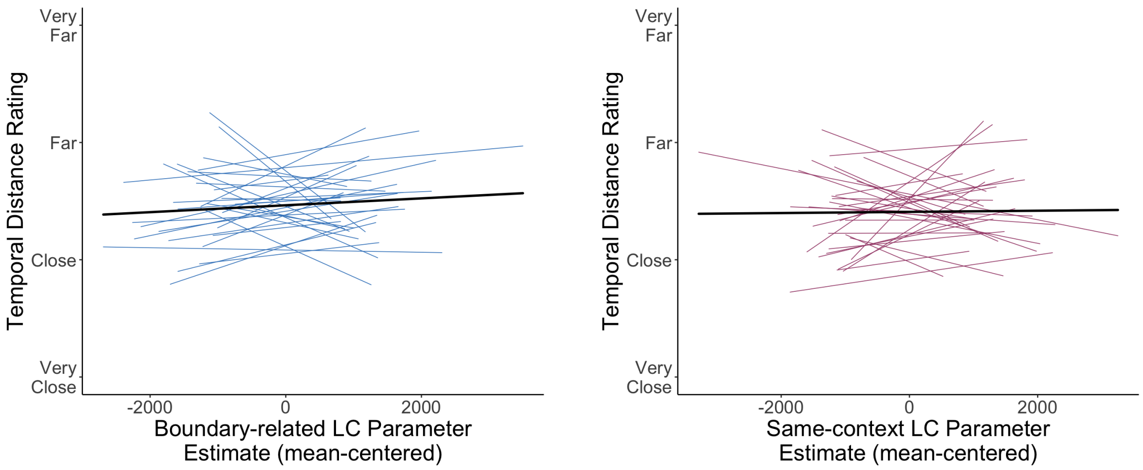

Supplementary Figure 6. Tone-related LC activation did not predict subjective temporal distance memory between item pairs. (Left panel) Participant-level trendlines plotting the null association between boundary-related LC parameter estimates (mean-centered) and temporal distance memory ratings (displayed as a continuous variable). (Right panel) Participant-level trendlines plotting the null association between same-context LC parameter estimates and temporal distance memory ratings. Dark, bold lines represent the average linear trend across participants. *n* = 32 participants. Statistical significance was tested using trial-level cumulative link models in which pair position at encoding was also modeled as an integer fixed-effect predictor. Source data are provided as a Source Data file.

Testing the relationship between tone-related VTA activation and temporal order memory. To determine whether temporal order accuracy could also be predicted by VTA activation, we fit a generalized linear mixed effects model that included tone-related VTA activation as a fixed-effect predictor of order accuracy (correct = 1, incorrect = 0). Pair position at encoding was modeled as an integer fixed-effect predictor. We found no significant main effect of VTA activation (z = 1.05, p = .30; odds ratio = 1.04, 95% CI of odds ratio = [.97, 1.11]) or VTA-by-pair type interaction effect (z = .40, p = .69; odds ratio = 1.01, 95% CI of odds ratio = [.95, 1.09]) on order accuracy (**Supplementary Figure 7**).

Comparing relationships between tone-related LC vs. VTA activation and order memory. Next, we clarified whether the coupling between brainstem activation and temporal order ratings for boundary pairs was specific to the LC. We performed a likelihood-ratio test that compared a full model with both VTA and LC activation as predictors with a restricted model that averaged LC and VTA activation as one predictor. To avoid a singular fit, these models were fit with a fixed effect representing the side of the screen containing the correct answer (i.e., left or right). The test revealed that the full model significantly improved fit over the restricted model ($\chi^2(1)$ = 9.21, p = .0024). This finding suggests that the relationship between brain activation and temporal order memory for boundary-spanning pairs was specific to the LC.

Comparing relationships between temporal order vs. distance memory and VTA activation. To compare the magnitude of the relationship between tone-related VTA activation and temporal memory for boundary pairs, we compared the standardized coefficients for VTA activation between the distance and order memory models. To avoid a singular fit, the order memory model was fit with a fixed effect representing the side of the screen containing the correct answer (i.e., left or right). The standardized coefficient for VTA activation in the distance memory model ($ß$ = .10, 95% CI = [.01, .20]) was not significantly different than in the order memory model ($ß$ = .05, 95% CI = [-.05, .16]). Thus, while VTA activation did not relate to temporal order memory, we cannot conclude that VTA coupling with memory was unique to temporal distance memory.

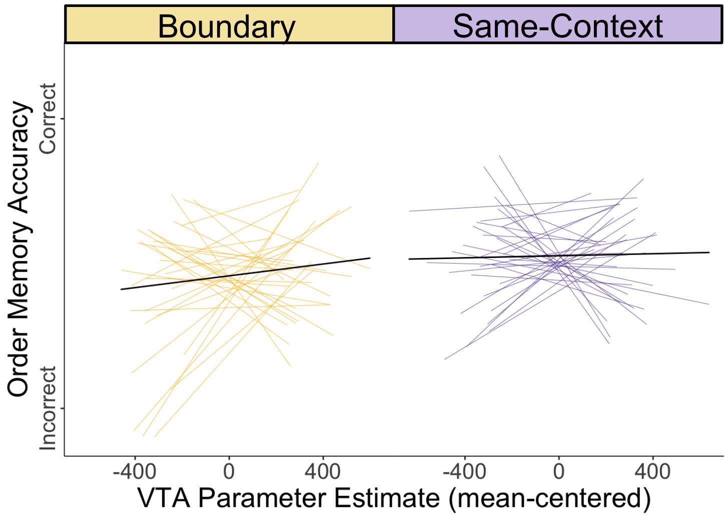

Supplementary Figure 7. Tone-related VTA activation did not predict temporal order memory accuracy. Participant-level trendlines plotting the null relationship between VTA parameter estimates (mean-centered) and order memory accuracy for boundary-spanning pairs (yellow; left) and same-context pairs (purple; right). For modeling, VTA parameter estimates were mean-centered for each condition separately. Order memory accuracy is displayed as a continuous variable. Dark, bold lines represent the average linear trend across participants. n = 32 participants. Statistical significance was tested using a generalized linear mixed effects model in which pair position at encoding was also modeled as an integer fixed-effect predictor. Source data are provided as a Source Data file.

Testing the relationship between blinking and temporal order memory. To determine whether temporal order accuracy could also be predicted by blinking, we fit a generalized linear mixed effects model that included extended blink count as a fixed-effect predictor of order accuracy (correct = 1, incorrect = 0). We found no significant main effect of extended blink count (z = -.55, p = .58; odds ratio = 1.00, 95% CI of odds ratio = [.99, 1.01]) or blink count-by-pair type interaction effect (z = -1.18, p = .24; odds ratio = 1.00, 95% CI of odds ratio = [.99, 1.00]) on order accuracy (**Supplementary Figure 8**).

Comparing relationships between temporal order vs. distance memory and blinking. To compare the magnitude of the relationship between blinking and temporal memory ratings for boundary pairs, we compared the standardized coefficients for blinking between the distance and order memory models. To avoid a singular fit, the order memory model was fit with a fixed effect representing the side of the screen containing the correct answer (i.e., left or right). The standardized coefficient for blinking in the distance memory model ($ß$ = .16, 95% CI = [.06, .27]) was significantly greater than in the order memory model ($ß$ = -.07, 95% CI = [-.18, .04]). This finding suggests that the relationship between blinking and memory for boundary-spanning pairs was greater for temporal distance memory than order memory.

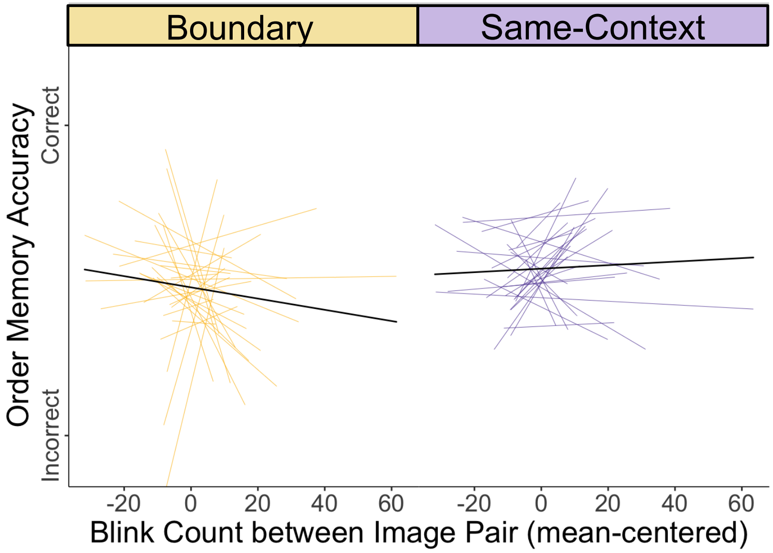

Supplementary Figure 8. Sustained blinking between to-be-tested item pairs did not predict memory accuracy for the temporal order of those items. Participant-level trendlines plotting the null relationship between extended blink count (mean-centered) and order memory accuracy for boundary-spanning pairs (yellow; left) and same-context pairs (purple; right). For modeling, blink counts were mean-centered for each condition separately. Order memory accuracy is displayed as a continuous variable. Dark, bold lines represent the average linear trend across participants. n = 28 participants. Statistical significance was tested using a generalized linear mixed effects model. Source data are provided as a Source Data file.

Testing the relationship between tone-related LC activation and blinking. To assess whether blinking behavior could also be predicted by LC activation, we fit linear mixed effects models. First, we examined local blink count. We found no significant main effect of LC activation on post-tone blink count (t(7308.17) = -1.44, p = .15; ß = -.02, 95% CI = [-.05, .0084]) (**Supplementary Figure 9, left panel**). There was also no significant LC-by-pair type interaction effect on blinking (t(7308.32) = -.97, p = .33; ß = -.02, 95% CI = [-.05, .02]).

Next, we examined temporally extended blink count between to-be-tested image pairs. We found no significant main effect of LC activation on extended blink count (t(2806) = 1.00, p = .32; ß = .01, 95% CI = [-.01, .04]) (**Supplementary Figure 9, right panel**). There was also no significant LC-by-pair type interaction effect (t(2806) = .90, p = .37; ß = .01, 95% CI = [-.01, .03]).

Comparing relationships between tone-related VTA vs. LC activation and blinking. Next, we clarified whether the coupling between brainstem activation and blinking between to-be-tested pairs was specific to the VTA. We performed a linear hypothesis test that compared a full model with both VTA and LC activation as predictors with a restricted model in which the VTA and LC coefficients were equal. The test revealed that these coefficients were not equal (χ^2(1) = 6.95, p = .0084), suggesting that the relationship between brain activation and temporally extended blink count was specific to the VTA.

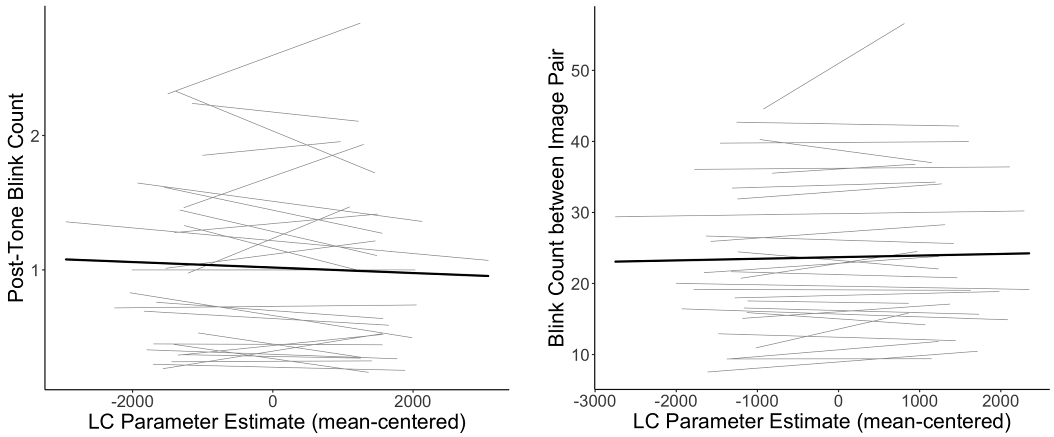

Supplementary Figure 9. Tone-related LC activation did not predict momentary or temporally extended changes in blink behavior. (Left panel) Participant-level trendlines plotting the null relationship between LC parameter estimates and post-tone blink count. (Right panel) Participant-level trendlines plotting the null relationship between LC parameter estimates and extended blink count. Dark, bold lines represent the average linear trend across participants. LC parameter estimates were mean-centered for each model separately. n = 28 participants. Statistical significance was tested using linear mixed effects models. Source data are provided as a Source Data file.

Hippocampal pattern similarity fMRI analyses. Left and right hippocampal subfields CA2/3, dentate gyrus (DG), and CA1 were segmented from each participant's high-resolution anatomical scan using Freesurfer 6.0 (https://surfer.nmr.mgh.harvard.edu/). To quality control this segmentation, we used guidelines designed for the Enhancing Neuro Imaging Genetics through Meta-Analysis (ENIGMA) consortium (https://enigma.ini.usc.edu/ongoing/enigma-hippocampal-subfields/). Seven participants were excluded in this process; therefore, the final sample size for these analyses was 25 participants. Validated hippocampal ROIs were then co-registered to each participant's native/run-specific functional space and thresholded at 0.2 to reduce spatial overlap between adjacent subfields.

For each of these hippocampal ROIs, we extracted activation patterns from the trial-unique beta maps produced by the LSS GLM, which modeled stimulus-specific activation patterns for all tones and images in a sequence. Here, we focused on the multivoxel patterns evoked by the image pairs as an index of hippocampal pattern stability across encoding, with more similar patterns reflecting representational stability and more dissimilar patterns reflecting temporal pattern separation. Hippocampal subfield pattern similarity scores were computed at the item pair level by correlating multivoxel patterns between each of the to-be-tested trial pairs from encoding. For more details, see Clewett et al. (2025).

Testing the relationship between boundary-related VTA activation and hippocampal pattern similarity across time. To determine whether VTA activation at boundaries predicted hippocampal pattern similarity for to-be-tested item pairs spanning those same boundaries, we fit a linear mixed effects model with six predictors of VTA activation at boundaries: left and right DG, CA1, and CA2/3 pattern similarity. Unlike the LC (Clewett et al., 2025), left DG pattern similarity did not significantly predict tone-induced VTA activation ($t(1066.28) = -.46$, $p = .65$; $ß = -.02$, 95% CI = [-.08, .05]). There were also no significant main effects of right DG, left or right CA1, or left or right CA2/3 (ps > .05) (all subfield results are displayed in **Supplementary Figure 10**).

Comparing relationships between boundary-related LC vs. VTA activation and hippocampal pattern similarity. To directly compare the effect of left DG pattern similarity on boundary-related LC and VTA activation, we added brain region (LC vs. VTA) as an interaction term in the model. We observed a significant region-by-left DG interaction effect ($t(2163.59) = -2.84$, $p = .0045$; $ß = -.07$, 95% CI = [-.11, -.02]). A simple slopes analysis revealed that the slopes of left DG pattern similarity significantly differed by brain region (t ratio = -2.84, p = .0045), such that LC activation at boundaries was more strongly linked to left DG pattern similarity ($b = -729.9$) than VTA activation ($b = -28.2$). There were no other significant interaction effects (ps > .05).

In summary, these findings suggest that momentary increases in VTA activation at boundaries did not modulate pattern similarity in hippocampal subfields. We also found that LC activation (vs. VTA) at boundaries was more strongly coupled with left DG pattern similarity for items spanning those boundaries, suggesting the LC may play a larger role in differentiating memory representations across time. Moreover, LC activation was selectively coupled with impairments in temporal order memory across boundaries, suggesting that the noradrenergic system might specifically modulate objective features of temporal memory.

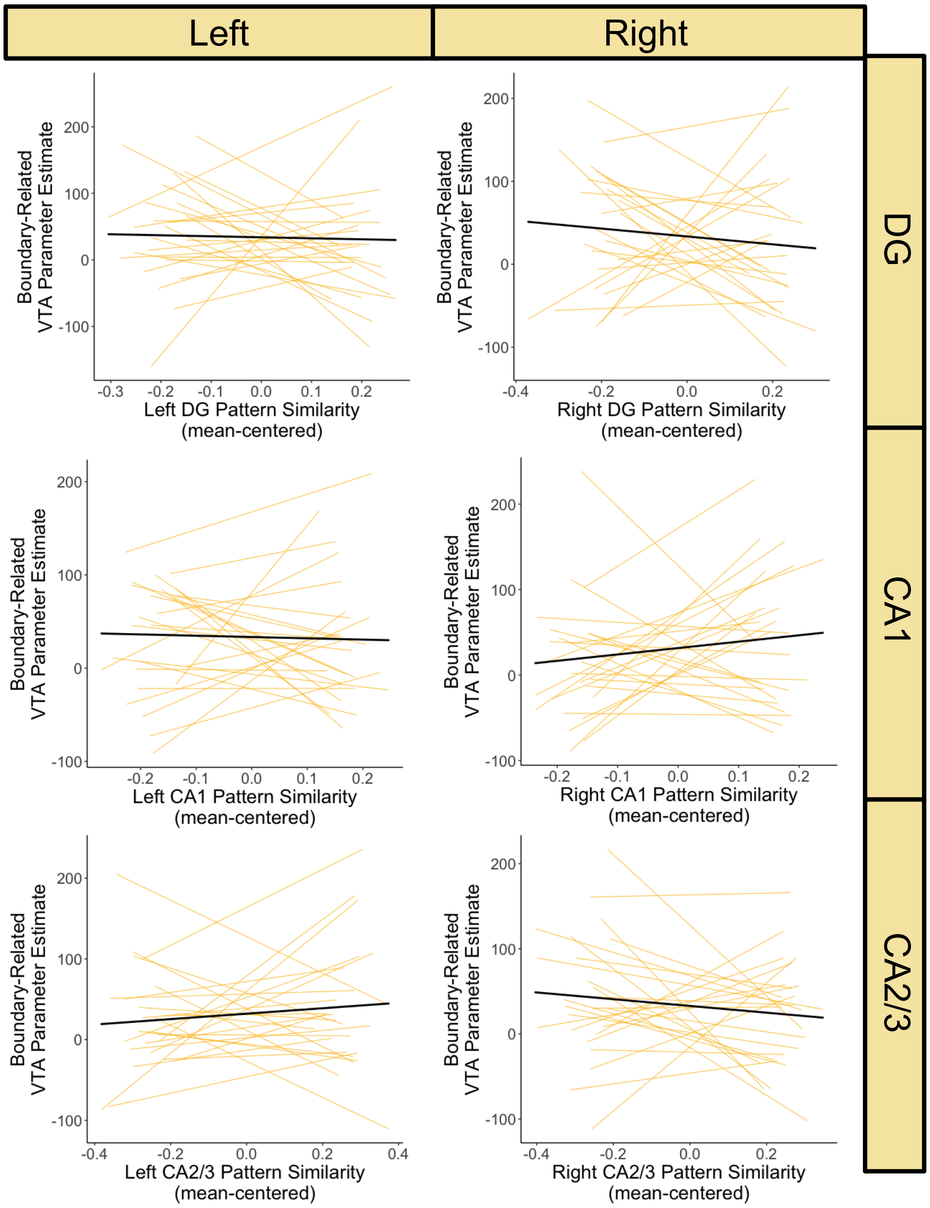

Supplementary Figure 10. Hippocampal pattern similarity for item pairs, including in the dentate gyrus, does not predict boundary-related VTA activation. Participant-level trendlines plotting the null relationships between hippocampal subfield pattern similarity for to-be-tested item pairs (in dentate gyrus, or DG, CA1, and CA2/3; left and right) and boundary-related VTA parameter estimates between those same item pairs. Dark, bold lines represent the average linear trend across participants. *n* = 25 participants. Each subfield's pattern similarity values were mean centered separately and were all included as predictors of boundary-related VTA activation in the same linear mixed effects model. Source data are provided as a Source Data file.

Pupil dilation temporal principal component analysis (PCA). Prior work shows that context shifts elicit increased pupil dilation, suggesting that boundaries engage central arousal processes (Clewett et al., 2025). Pupil dilation, however, is complex and is mediated by multiple autonomic pathways and neuromodulatory systems (Reimer et al., 2016). Building on earlier work (Clewett et al., 2020), we use a temporal principal component analysis (PCA) to decompose boundary-related pupil dilations into its distinct temporal features, providing a unique opportunity to link event segmentation to different neural and behavioral effects. The temporal PCA on tone-evoked pupil dilations revealed three canonical pupil components identified in prior work, including a biphasic response that may index separate influences of parasympathetic and sympathetic nervous system regulation on pupil diameter (Clewett et al., 2020; Steinhauer and Hakerem, 1992). The temporal characteristics of these pupil components, including their latencies-to-peak and percent of explained variance, were as follows: (1) an early-peaking component (684 ms; 89.26% variance); (2) intermediate-peaking component (1,420 ms; 8.40% variance); and (3) slowly decreasing component (19.6 ms; 1.27% variance). For more details about these methods and results, see Clewett et al. (2025).

Testing the relationship between boundary-related VTA activation and three distinct temporal features of tone-evoked pupil dilation. In previously published work using this dataset, we found that pupil components #2 and #3 were both positively coupled with boundary-related LC activation (Clewett et al., 2025). Here, we tested whether the pupil components were also correlated with engagement of the VTA. Using Spearman's rho correlations, we found that boundary-induced VTA activation was not significantly correlated with boundary-induced loading on pupil component #1 (ρ = .14, p = .47), pupil component #2 (ρ = .11, p = .58), or component #3 (ρ = -.079, p = .69; **Supplementary Figure 11**).

Comparing the relationships between boundary-related VTA vs. LC activation and pupil dilation. Next, we examined whether the coupling between brainstem activation and the three pupil components was specific to the LC using a Steiger's Z test. We found no significant differences in the linear relationships between LC and VTA activation and pupil component #1 (z = -.44, p = .66), pupil component #2 (z = -.44, p = .66), or pupil component #3 (z = .48, p = .63). Thus, while VTA activation did not relate to distinct temporal characteristics of pupil dilation at boundaries, we cannot conclude that neuromodulatory coupling with pupil responses was unique to LC activation.

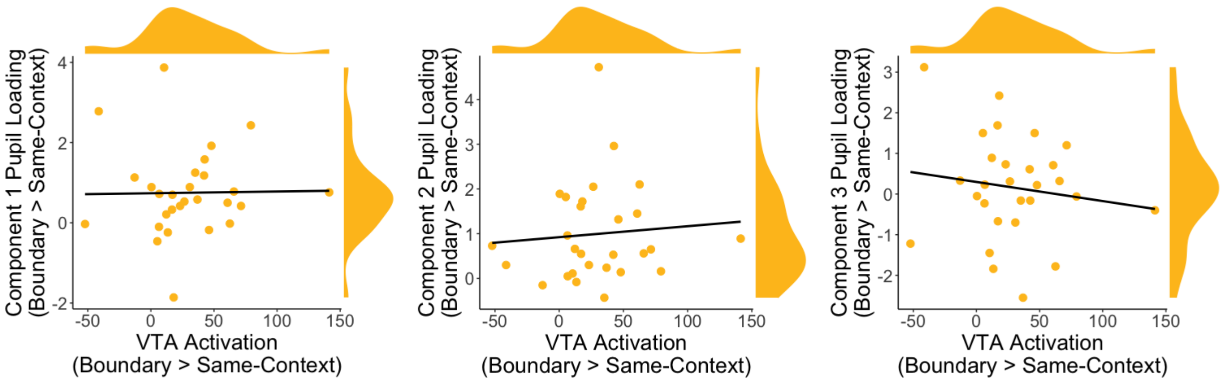

Supplementary Figure 11. Boundary-related VTA activation was not associated with any of three temporal features of tone-evoked pupil dilations. Spearman's rho correlation plots showing that boundary-induced VTA activation was not significantly correlated with boundary-related engagement of pupil dilation component #1 (left panel), component #2 (middle panel), or component #3 (right panel). Individual dots represent each participants' data (n = 28 participants). *X*- and *y*-distributions also displayed. Source data are provided as a Source Data file.

Identifying which blinking periods and types of stimulus-elicited blinked predicted time dilation in memory. In this set of analyses, we aimed to identify the specific blink periods and stimuli that predict later distortions in temporal distance memory.

Average patterns of blinking across the inter-pair windows. To capture temporally dynamic patterns of blinking across the event sequence, we first divided each inter-item window into two coarse-grained intervals: 1) the interval before the reference tone (*M* duration = 12.28s); and 2) the interval *after* the reference tone (including that tone; *M* duration = 20.87s). For more details, see **Supplementary Figure 12** below. On average, participants blinked 8.83 times before the reference tone (*M* boundary pairs = 8.78 blinks; *M* same-context pairs = 8.87 blinks) and 15.43 times after the reference tone (*M* boundary pairs = 15.77 blinks; *M* same-context pairs = 15.10 blinks).

Next, given that the interval after the reference tone carried key contextual information in this task (e.g., that the auditory-task context had or had not changed), we divided this interval further. Specifically, we identified even more fine-grained intervals associated with each stimulus: 1) images; and 2) tones (**Supplementary Figure 12**). We observed that most blinks occurred after images (*M* overall = 12.56 blinks across images; *M* boundary pairs = 12.77 blinks; *M* = same-context pairs = 12.34 blinks) compared to tones (*M* overall = 4.65 blinks across tones; *M* boundary pairs = 4.76 blinks; *M* = same-context pairs = 4.54 blinks). This is a sensible result, given that images have a longer duration and subsequent ISI compared to tones.

Relating specific blink periods to temporal distance memory. Here, we conducted linear mixed-effects models to test which stimulus-evoked blinks and periods of blinking were significantly predictive of temporal distance ratings.

Blinks in the earlier vs. later interval did not differentially influence temporal memory predictions. First, we tested whether the interval before versus after the reference tone drove this effect. There was no significant interaction between blink count, Interval Type (before vs. after reference tone), and Pair Type (boundary vs. same-context) on distance memory ($z = .45$, $p = .65$; odds ratio = 1.00, 95% CI of odds ratio = [1.00, 1.01]).

Focusing on the later interval, blinking after a tone switch vs. no switch predicted greater time dilation in memory. While the prior interaction effects were null, we next focused specifically on the blink window *after* the reference tone, because this carried the critical information about the auditory-task context, In this period, we found a significant interaction between blink count and Pair Type on distance memory ($z = 2.12$, $p = .034$; odds ratio = 1.01, 95% CI of odds ratio = [1.00, 1.02]). To break down this interaction effect, we then examined the two Pair Types separately. For boundary pairs, there was a marginally significant main effect of post-tone switch blink count on distance memory ($z = 1.87$, $p = .06$; odds ratio = 1.01, 95% CI of odds ratio = [1.00, 1.02]), such that more blinking after the tone switch predicted greater subsequent time dilation between the to-be-tested object pairs. In contrast, for same-context pairs, there was no significant effect of post-reference tone blink count on distance memory ($z = -1.04$, $p = .30$; odds ratio = .99, 95% CI of odds ratio = [.98, 1.01]). Therefore, blinks after the reference tone predicted later time dilation only when there had been a tone switch, denoting a new auditory context.

Investigating whether image- or tone-evoked blink effects in the later inter-pair interval predicted time dilation in memory. Zooming in on this later interval further, we asked whether stimulus type mattered; that is, whether blinks following either *images* or *tones* were the driving factor behind changes in temporal distance ratings. We found that there were no interaction effects between blink count, Stimulus Type (Images vs. Tones), and Pair Type on distance memory (all *p*s > .05). Thus, at least during the critical latter part of the inter-pair interval, blinks following images vs. tones did not relate to temporal distance memory.

Given the lack of an interaction effect and that tones carried the important contextual information during the task, we next focused on tone-related blinking alone in the later inter-pair interval. We found a significant interaction between Pair Type and total blink count following tones on distance memory (z = 2.63, p = 0.0086; odds ratio = 1.03, 95% CI of odds ratio = [1.01, 1.05]). To break down this interaction effect, we analyzed the two Pair Types separately. For boundary pairs, there was a significant main effect of total blink count following tones on distance memory (z = 2.11, p = .035; odds ratio = 1.04, 95% CI of odds ratio = [1.00, 1.07]), such that more blinking after the tone switch predicted greater time dilation between the to-be-tested object pairs. In contrast, for same-context pairs, there was no significant effect of total blink count following tones on distance memory (z = -1.56, p = 0.12; odds ratio = .98, 95% CI of odds ratio = [.95, 1.01]). Together, these findings suggest that the coupling between blinking and time dilation in memory was driven by tone switches, the stimulus that carried the critical signal denoting an event boundary.

In summary, our analyses showed that blinks following a tone switch (versus no switch) predicted greater time dilation in memory between the to-be-tested object pairs. This link between blinking and memory distortion was related to the occurrence of tones, suggesting that event boundaries play an important role in triggering dopaminergic processes that shape later memory separation effects.

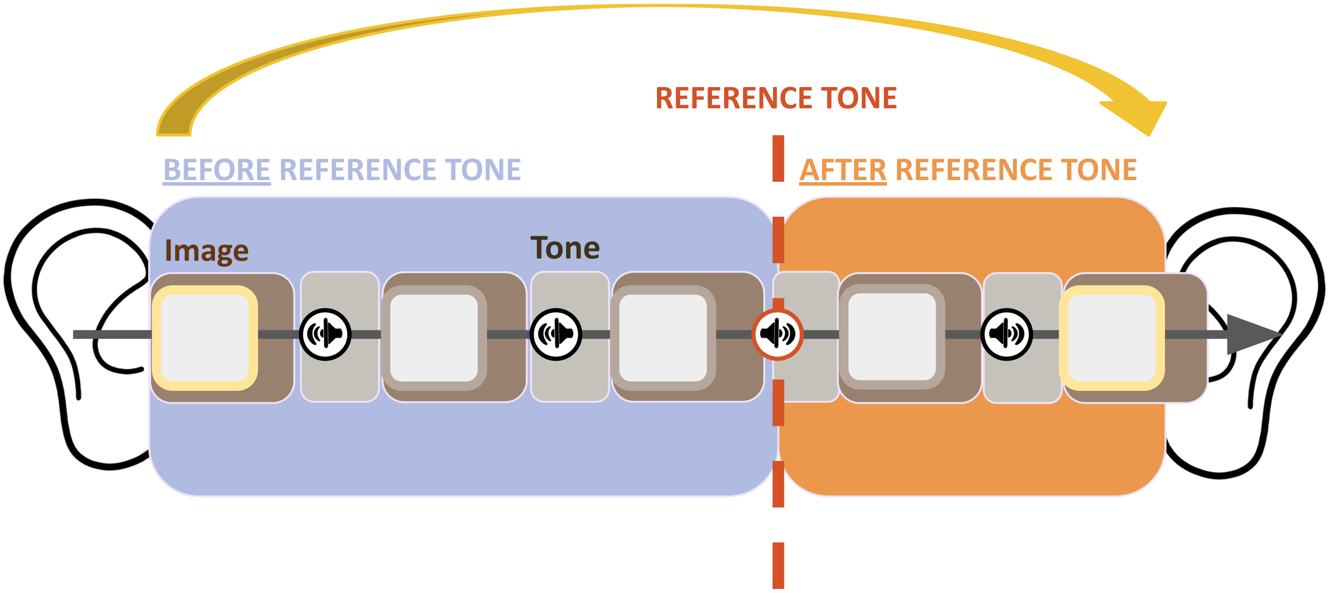

Supplementary Figure 12. Dividing the window between to-be-tested pairs into specific intervals of interest. Example intervening window for each to-be-tested image pair (see yellow arrow connecting pair of squares). Each window was approximately 32.5s long, containing 5 images and 4 tones total. These windows can be divided into coarse- and fine-grained intervals of interest. The coarse-grained intervals are separated by the reference tone (red dashed line), which is the tone of interest between each to-be-tested pair. For boundary-spanning pairs (example shown here), this was the tone switch that denoted an event boundary. For same-context pairs, this was a position-matched tone (for more details, see **Supplementary Figure 1, bottom panel**). Therefore, the two resulting intervals are as follows: (1) Before the reference tone (blue), from the onset of the first to-be-tested image to the onset of the reference tone; and (2) After the reference tone (orange), from the onset of the reference tone to the offset of the second to-be-tested image. Additionally, the window can be segmented further into fine-grained intervals that are associated with each individual stimulus: (1) Image intervals (brown), from the onset of the image to the end of the subsequent ISI (2.5s + variable + 0.5s); and (2) Tone intervals (gray), from the onset of the tone to the end of the subsequent ISI (variable). The final image interval (brown) extends partially outside of the "After the reference tone" (orange) interval, as this image interval also contains the ISI after the final image offset.

Supplemental References

1. Clewett, D., Gasser, C., & Davachi, L. (2020). Pupil-linked arousal signals track the temporal organization of events in memory. *Nature Communications*, *11*(1), 4007.
2. Clewett, D., Huang, R., & Davachi, L. (2025). Locus coeruleus activation “resets” hippocampal event representations and separates adjacent memories. *Neuron*.
3. Pu, Y., Kong, X. Z., Ranganath, C., & Melloni, L. (2022). Event boundaries shape temporal organization of memory by resetting temporal context. *Nature Communications*, *13*(1), 622.
4. Reimer, J., McGinley, M. J., Liu, Y., Rodenkirch, C., Wang, Q., McCormick, D. A., & Tolias, A. S. (2016). Pupil fluctuations track rapid changes in adrenergic and cholinergic activity in cortex. *Nature Communications*, *7*(1), 13289.
5. Steinhauer, S. R. & Hakerem, G. The pupillary response in cognitive psychophysiology and schizophrenia. *Annals of the New York Academy of Sciences* 658, 182–204 (1992).
