# Supplementary material for: Dopaminergic processes predict temporal distortions in event memory
Source: Nat Commun. 2026 Mar 14;17:3971. doi: 10.1038/s41467-026-69950-8 (PMC13133266; doi:10.1038/s41467-026-69950-8)
Supplement: Supplementary file 2 — Reporting Summary [file 41467_2026_69950_MOESM2_ESM.pdf]

nature portfolio

Corresponding author(s): David Clewett

Last updated by author(s): Jan 30, 2026

Reporting Summary

Nature Portfolio wishes to improve the reproducibility of the work that we publish. This form provides structure for consistency and transparency in reporting. For further information on Nature Portfolio policies, see our Editorial Policies and the Editorial Policy Checklist.

Statistics

For all statistical analyses, confirm that the following items are present in the figure legend, table legend, main text, or Methods section.

n/a	Confirmed	
☐	☒	The exact sample size (*n*) for each experimental group/condition, given as a discrete number and unit of measurement
☐	☒	A statement on whether measurements were taken from distinct samples or whether the same sample was measured repeatedly
☐	☒	The statistical test(s) used AND whether they are one- or two-sided<br>*Only common tests should be described solely by name; describe more complex techniques in the Methods section.*
☐	☒	A description of all covariates tested
☐	☒	A description of any assumptions or corrections, such as tests of normality and adjustment for multiple comparisons
☐	☒	A full description of the statistical parameters including central tendency (e.g. means) or other basic estimates (e.g. regression coefficient) AND variation (e.g. standard deviation) or associated estimates of uncertainty (e.g. confidence intervals)
☐	☒	For null hypothesis testing, the test statistic (e.g. *F*, *t*, *r*) with confidence intervals, effect sizes, degrees of freedom and *P* value noted<br>*Give P values as exact values whenever suitable.*
☒	☐	For Bayesian analysis, information on the choice of priors and Markov chain Monte Carlo settings
☐	☒	For hierarchical and complex designs, identification of the appropriate level for tests and full reporting of outcomes
☒	☐	Estimates of effect sizes (e.g. Cohen's *d*, Pearson's *r*), indicating how they were calculated

Our web collection on statistics for biologists contains articles on many of the points above.

Software and code

Policy information about availability of computer code

Data collection	Custom E-Prime (v3.0) code was used to build the experiment and collect the data in this study.
Data analysis	FSL (v6.0) and Freesurfer (v6.0) software, in addition to ENIGMA quality control code (https://enigma.ini.usc.edu/ongoing/enigma-hippocampal-subfields/), were used in processing and analyzing the fMRI data in this study. Custom MATLAB (R2022b) code was used in processing and analyzing the blink data in this study. Custom RStudio (v2024.2.29) code was used in cleaning and analyzing all transformed data in this study. This RStudio code is available on the first author's OSF profile (https://osf.io/yt6hm).

For manuscripts utilizing custom algorithms or software that are central to the research but not yet described in published literature, software must be made available to editors and reviewers. We strongly encourage code deposition in a community repository (e.g. GitHub). See the Nature Portfolio guidelines for submitting code & software for further information.

Data

Policy information about availability of data

All manuscripts must include a data availability statement. This statement should provide the following information, where applicable:

- Accession codes, unique identifiers, or web links for publicly available datasets
- A description of any restrictions on data availability
- For clinical datasets or third party data, please ensure that the statement adheres to our policy

The data generated in this study is deposited on the first author's OSF profile (https://osf.io/yt6hm).

Research involving human participants, their data, or biological material

Policy information about studies with human participants or human data. See also policy information about sex, gender (identity/presentation), and sexual orientation and race, ethnicity and racism.

Reporting on sex and gender	32 participants were included in the current study (20 female). Neither sex nor gender were included as fixed effects or covariates in the current analyses because they were not variables of interest in this study.
Reporting on race, ethnicity, or other socially relevant groupings	Of our 32 participants, 15 identified as Asian, 2 as Black/African American, 4 as more than one race, and 11 as White. These racial identifications were self-reported by each participant, chosen from options provided on a standard demographic questionnaire.
Population characteristics	See above.
Recruitment	Participants were recruited from the New York University (NYU) Psychology Subject Pool and the local community. Eligibility criteria included: (1) normal or corrected-to-normal vision and hearing; (2) no metal in body; (3) not currently taking beta-blocker medications; and (4) not currently taking psychoactive medications.
Ethics oversight	All experimental protocols were approved by the New York University Institutional Review Board.

Note that full information on the approval of the study protocol must also be provided in the manuscript.

Field-specific reporting

Please select the one below that is the best fit for your research. If you are not sure, read the appropriate sections before making your selection.

☐ Life sciences ☒ Behavioural & social sciences ☐ Ecological, evolutionary & environmental sciences

For a reference copy of the document with all sections, see nature.com/documents/nr-reporting-summary-flat.pdf

Behavioural & social sciences study design

All studies must disclose on these points even when the disclosure is negative.

Study description	Quantitative experimental
Research sample	Thirty-two healthy young adult participants (mean age 22 yr old, SD 2.7 yr; 20 female) were recruited from the New York University Psychology Subject Pool and the local community. This sample was recruited to represent healthy young adults, and was generally representative of this population (although it consisted mostly of female college students, who tend to be overrepresented in psychology research). Eligibility criteria included: (1) normal or corrected-to-normal vision and hearing; (2) no metal in body; (3) not currently taking beta-blocker medications; and (4) not currently taking psychoactive medications.
Sampling strategy	To estimate the total number of participants needed in this study, we used a G*Power 3.1 power analysis (alpha = 0.05, power = 0.80, d = .80) based on the effect size derived from the pooled temporal memory performance from a very similar event boundary experiment (Clewett, Gasser, & Davachi, 2020). This power analysis indicated a sample size of 29. However, to account for attrition, we recruited 36 participants. We used convenience sampling (i.e., expressed interest from flyer advertisements) to recruit these participants.
Data collection	All data were collected in-person at an MRI scanner facility at New York University. Only the researcher (D. C.) and participant were present. Participants completed a modified version of an event sequence encoding task, an arrow distractor task, and temporal memory tests (Clewett et al., 2020). The researcher was not blinded to experimental condition and/or the study hypothesis, given that all participants received both conditions (within-subjects design) and the paradigm was administered objectively using custom E-Prime code within the MRI scanner.
Timing	Data were collected in-person between December 12, 2018 and June 27, 2019.
Data exclusions	Four participants were fully excluded from analyses (reasons included falling asleep in the scanner and audio malfunction), resulting in a final sample size of 32 participants. Of these 32 participants, five requested to leave the scanner early and thus did not complete all 10 blocks of the task (n = 2 completed 9 blocks, n = 1 completed 8 blocks, n = 2 completed 7 blocks).<br><br>Four participants' eye tracking data was excluded due to equipment malfunction or poor quality, leaving 28 participants with usable data for all analyses that included blink data.<br><br>We excluded the first same-context item pair from all analyses, because this pair contained the first image in each block. As such, it likely constituted a task-irrelevant event boundary and would therefore produce different behavioral effects than the other same-context pairs. Due to a programming error, one of the boundary pairs from each list also contained an incorrect item. Data for these specific pairs (appearing once per block across 23 participants) were excluded from the analyses. Finally, one block was excluded for 9 participants due to a timing error.<br><br>Entire blocks with excessive head motion overall (conservatively defined as MRI mean frame displacement > 1mm) were excluded from analysis, resulting in the removal of one block each from three participants.

We excluded noisy datapoints using boxplot outlier removal by-participant, resulting in the removal of approximately 3% of the tone-related VTA activation trials across the entire dataset. To acquire highly conservative estimates of blink behavior that were not confounded by noise or data loss, blink intervals with more than 25% invalid samples were excluded from analyses. As with the VTA data, we also cleaned the trial-level blink responses using boxplot outlier detection within each participant. After these exclusions, approximately 78% of local blink intervals remained per participant, on average. For extended blink counts between to-be-tested item pairs, we conducted the same noise-related and by-participant boxplot outlier removal procedure, which left approximately 79% of blink windows per participant, on average.

Non-participation

Five participants requested to leave the scanner early and thus did not complete all 10 blocks of the task (n = 2 completed 9 blocks, n = 1 completed 8 blocks, n = 2 completed 7 blocks). Four participants fully dropped out of the study.

Randomization

Participants were not allocated into separate groups because all factors were within-subjects in this study.

Reporting for specific materials, systems and methods

We require information from authors about some types of materials, experimental systems and methods used in many studies. Here, indicate whether each material, system or method listed is relevant to your study. If you are not sure if a list item applies to your research, read the appropriate section before selecting a response.

Materials & experimental systems

n/a	Involved in the study
☒	☐ Antibodies
☒	☐ Eukaryotic cell lines
☒	☐ Palaeontology and archaeology
☒	☐ Animals and other organisms
☒	☐ Clinical data
☒	☐ Dual use research of concern
☒	☐ Plants

Methods

n/a	Involved in the study
☒	☐ ChIP-seq
☒	☐ Flow cytometry
☐	☒ MRI-based neuroimaging

Plants

Seed stocks

N/A

Novel plant genotypes

N/A

Authentication

N/A

Magnetic resonance imaging

Experimental design

Design type

Event-related task design

Design specifications

There were 10 blocks of the event sequence encoding task. In each block, there were 32 trials during which a neutral object image was presented for 2.5s. Between each trial, there was a jittered ISI lasting either 3, 5, or 7s. At the midpoint of the ISI, a 1s pure tone was presented in either the left or right ear.

After each block of the sequence encoding task, participants completed a brief arrow detection task (45s). Participants were presented with 0.5s left-facing or right-facing arrows in the center of the screen, separated by 0.5s ISIs of fixation.

After the arrow detection task, participants completed two temporal memory tests: the first for temporal order and the second for temporal distance. In the temporal order memory test, participants judged which of two items appeared most recently in the prior sequence. Those results are reported elsewhere because they were not central to the hypotheses in the current study. For the temporal distance memory test (of interest in the current study), 14 item pairs from the encoding sequence were presented for a fixed duration of 5s each. Importantly, each of these item pairs had been separated by exactly three images during encoding. The jittered ISIs were pseudorandomized such that there was always approximately 32.5s from the onset of the first image in the to-be-tested pair to the offset of its pairmate.

Behavioral performance measures

For each image in the event encoding sequence task, participants made a button press to respond to a simple orienting

question ("Is this object larger or smaller than a standard shoebox?"). Any responses to this question were used to confirm engagement with the encoding task and measure by mean response times (reported elsewhere; Clewett et al., in press).

In the arrow detection task, participants were instructed to indicate which direction the arrow was facing via button press as quickly as possible. Any responses were used to confirm engagement with the task, which was meant to separate the encoding and retrieval tasks and reduce any recency effects in memory.

In the temporal distance memory test, participants were asked to judge how far apart in time two images appeared. There were four options: 'very close,' 'close,' 'far,' and 'very far.' These ratings measured subjective temporal distortions in memory across conditions, and were analyzed as an ordinal variable.

Acquisition

Imaging type(s)

Structural and functional

Field strength

3 Tesla

Sequence & imaging parameters

All neuroimaging data were acquired with 3T Siemens Magnetom PRISMA scanner using a 64-channel matrix head coil. First, participants underwent a high-resolution MPRAGE T1-weighted anatomical scan (slices = 240 sagittal; TR = 2300ms; TE = 2.32ms; TI = 900ms; FOV = 230 mm; voxel in-plane resolution = 0.9 mm2; slice thickness = 0.9 mm; flip angle = 6 degrees; bandwidth = 200 Hz/Px; GRAPPA with acceleration factor = 2; scan duration: 5m 21s).

Next, participants underwent a T2-weighted functional scan (slices = 240 sagittal; TR = 3200ms; TE = 564 ms; FOV = 230 mm; voxel in-plane resolution = 0.9 mm2; slice thickness = 0.9 mm; flip angle = 6 degrees; bandwidth = 200 Hz/Px; GRAPPA with acceleration factor = 2; scan duration: 3m 7s). Additionally, we collected two fieldmap scans to assist with functional imaging unwarping (1 in anterior-posterior (AP) phase encoding direction and 1 in posterior-anterior (PA) phase encoding direction). Afterwards, we collected a short fast spin echo (FSE) sequence MRI scan that enables visualization of the locus coeruleus (LC).

After the structural scans were collected, participants underwent separate functional imaging for each of the 10 encoding blocks and the 10 retrieval blocks. Functional scans were collected using a whole-brain T2*-weighted multiband echo planar imaging (EPI) sequence (128 volumes per encoding block; TR = 2000ms; TE = 28.6ms, voxel in-plane resolution = 1.5 x 1.5 mm2; slice thickness = 1 mm with no gap; flip angle = 75 degrees, FOV = 204 mm X 204 mm; 136 X 136 matrix; phase encoding direction: anterior-posterior; GRAPPA factor = 2; multiband acceleration factor = 2). In each volume, 58 slices were tilted -20 degrees of the anterior commissure-posterior commissure line and were collected in an interleaved order.

Area of acquisition

The ventral tegmental area (VTA) was our region of interest. To create an anatomical mask of the VTA, we used a publicly available probabilistic atlas that was originally created using individual hand-drawn ROIs (Murty et al., 2014). This standard-space VTA mask was thresholded at 75% probability to increase its spatial specificity and then registered into each participant's functional run (i.e., block) of the encoding task.

Diffusion MRI ☐ Used ☒ Not used

Preprocessing

Preprocessing software

Image preprocessing was performed using FSL Version 6.00 (FMRIB’s Software Library, www.fmrib.ox.ac.uk/fsl). Functional images were preprocessed using the following steps: removal of non-brain tissue using BET; B0 unwarping using fieldmap images; grand-mean intensity normalization of the 4D data set by a single multiplicative factor; and application of a high-pass temporal filter of 100s. No spatial smoothing was applied due to the small size of the VTA and to preserve spatial specificity.

Normalization

Each participant’s denoised mean functional volume was co-registered to their T1-weighted high-resolution anatomical image using brain-based registration (BBR). Anatomical images were then co-registered to the 2 mm isotropic MNI-152 standard-space brain using an affine registration with 12 degrees of freedom.

Normalization template

The 2 mm isotropic MNI-152 standard-space brain was used.

Noise and artifact removal

Motion correction was performed using the MCFLIRT tool, which produced six motion nuisance regressors. Additionally, fsl_motion_outliers was used to identify volumes with extreme head movements, or frame displacements, using the DVARS option. Both this matrix of outlier volumes and the six motion regressors were as modeled as covariates in the subsequent GLM analyses. Entire blocks with excessive head motion overall (conservatively defined as mean frame displacement > 1 mm) were excluded from analysis, resulting in the removal of one block each from three participants.

Eight separate physiological nuisance signal regressors were extracted for the subsequent GLM analyses. First, FSL FAST was used to decompose each participant’s high-resolution anatomical images into probabilistic tissue masks for white matter (WM), grey matter (GM), and cerebrospinal fluid (CSF). The CSF and WM masks were thresholded at 75% tissue-type probability to increase their spatial specificity and reduce potential overlap. Following a similar approach to Bartoň et al. (2019), we defined eight 4 mm spheres in representative regions of WM and CSF (four of each type; for exact coordinates, see Bartoň et al., 2019). The eight spheres and WM and CSF anatomical masks were then transformed into each participant or block’s native functional space and merged to further increase their spatial specificity. Nuisance timeseries for each of the four WM and four CSF merged masks were then extracted from each block’s preprocessed functional data and modeled as nuisance regressors in the GLMs.

Volume censoring

Entire blocks with excessive head motion overall (conservatively defined as mean frame displacement > 1 mm) were

excluded from analysis, resulting in the removal of one block each from three participants. Noisy values were flagged and removed from the analyses using the fsl_motion_outliers tool in FSL and the default DVARS filtering parameter.

Statistical modeling & inference

Model type and settings

To estimate VTA activation at the trial level, Least Squares Separate (LSS) GLM analyses were used on the unsmoothed functional data from the sequence encoding task.

Each LSS-GLM contained a total of 64 task-related regressors for each block of the encoding task, because there were 32 tones and 32 images in each block. Each tone was modeled as a 1s stick function and each image was modeled as 2.5s stick function. Both regressors were convolved with a dual-gamma hemodynamic response function (HRF). A total of 64 separate LSS-GLM analyses were conducted for each block of the ten blocks of the task, where one stimulus (image or tone) served as the regressor of interest and all other trials were modeled as a separate regressor. Each LSS-GLM thereby resulted in a unique activation estimate (i.e., beta map) across the whole brain for each stimulus (Mumford et al., 2012; Mumford, Davis, & Poldrack, 2014). To control for noise, a total of 14 nuisance regressors (4 WM, 4 CSF, and 6 motion regressors) were included in each GLM, along with individual nuisance regressors for trials with extreme head movements.

Effect(s) tested

To assess trial-level relationships between our key variables, we used linear mixed-effects and cumulative link modeling.

Fixed effects differed by model, and included tone type (boundary tone, same-context tone), pair type (boundary-spanning pair, same-context pair), pair position at encoding, trial-level VTA parameter estimate, post-tone blink count, and extended blink count.

In Supplementary Material, models of temporal order memory were fitted with either a fixed or random effect representing the side of the screen containing the correct answer (i.e., left or right). Specifically, 'correct side' was modeled as a fixed effect as needed to avoid a singular fit. Our model regarding hippocampal pattern similarity was fitted with fixed effects for left and right dentate gyrus, left and right CA1, and left and right CA2/3 pattern similarity. Other trial-level parameter estimates included those for the locus coeruleus and anterior cingulate cortex.

Each model included random intercepts for participant ID with fixed slopes.

Specify type of analysis: ☐ Whole brain ☒ ROI-based ☐ Both

Anatomical location(s)

The ventral tegmental area (VTA) was our region of interest. To create an anatomical mask of the VTA, we used a publicly available probabilistic atlas that was originally created using individual hand-drawn ROIs (Murty et al., 2014). This standard-space VTA mask was thresholded at 75% probability to increase its spatial specificity and then registered into each participant's functional run (i.e., block) of the encoding task.

Statistic type for inference

(See Eklund et al. 2016)

Cluster thresholding with a z threshold of 3.1 and a cluster p threshold of .05

Correction

Correction for family-wise error rate (FWE)

Models & analysis

n/a	Involved in the study
☒	☐ Functional and/or effective connectivity
☒	☐ Graph analysis
☐	☒ Multivariate modeling or predictive analysis

Multivariate modeling and predictive analysis

Left and right hippocampal subfields CA2/3, dentate gyrus (DG), and CA1 were segmented from each participant’s high-resolution anatomical scan using Freesurfer 6.0 (https://surfer.nmr.mgh.harvard.edu/). Validated hippocampal ROIs were then co-registered to each participant’s native/run-specific functional space and thresholded at 0.2 to reduce spatial overlap between adjacent subfields.

For each of these hippocampal ROIs, we extracted activation patterns from the trial-unique beta maps produced by the LSS GLM, which modeled stimulus-specific activation patterns for all tones and images in a sequence. Here, we focused on the multivoxel patterns evoked by the image pairs as an index of hippocampal pattern stability across encoding, with more similar patterns reflecting representational stability and more dissimilar patterns reflecting temporal pattern separation. Hippocampal subfield pattern similarity scores were computed at the item pair level by correlating multivoxel patterns between each of the to-be-tested trial pairs from encoding. For more details, see Clewett et al. (2025).
